# Four and a Half LIM Domains Protein 2 Mediates Bortezomib-Induced Osteogenic Differentiation of Mesenchymal Stem Cells in Multiple Myeloma Through p53 Signaling and β-Catenin Nuclear Enrichment

**DOI:** 10.3389/fonc.2021.729799

**Published:** 2021-09-13

**Authors:** Zhenqing Xie, Yan Xu, Xiaojing Wei, Gang An, Mu Hao, Zhen Yu, Lugui Qiu

**Affiliations:** ^1^State Key Laboratory of Experimental Hematology, National Clinical Research Center for Blood Diseases, Institute of Hematology & Blood Diseases Hospital, Chinese Academy of Medical Sciences and Peking Union Medical College, Tianjin, China; ^2^Department of Laboratory Medicine, The First Affiliated Hospital of Fujian Medical University, Fuzhou, China

**Keywords:** FHL2, bortezomib, osteogenic differentiation, multiple myeloma, p53, β-catenin, mesenchymal stem cells

## Abstract

Myeloma bone disease (MBD), caused by the inhibition of osteoblast activity and the activation of osteoclast in the bone marrow environment, is the most frequent and life-threatening complication in multiple myeloma (MM) patients. Bortezomib (Bzb) was shown to promote MM-derived mesenchymal stem cells (MM-MSCs) differentiation to osteoblast *in vitro* and in animal models, promoting the bone formation and regeneration, may be mediated *via* β-catenin/T-cell factor (TCF) pathway. Further defining molecular mechanism of Bzb-enhanced bone formation in MM will be beneficial for the treatment of myeloma patients. The present study has identified for the first time four and a half LIM domains protein 2 (FHL2), a tissue-specific coregulator that interacts with many osteogenic marker molecules, as a therapeutic target to ameliorate MM bone disease. First, increased messenger RNA (mRNA) and protein levels of FHL2, and the mRNA level of main osteoblast markers (including Runx2, ALP, and Col1A1), were found in MM-patients-derived MSCs after Bzb treatment. FHL2 KD with short hairpin RNA (shRNA) reduced the expression of osteoblast marker genes and blocked the osteogenic differentiation of MM-MSCs regardless of the presence or absence of Bzb, implying that FHL2 is an important activator of the osteogenic differentiation of human MSCs under a proteasome inhibition condition. Molecular analysis showed that the enhanced expression of FHL2 was associated with the Bzb-induced upregulation of p53. No significant change at protein level of total β-catenin was observed with or without Bzb treatment. However, it was mostly enriched to nuclei in MSCs after Bzb treatment. Moreover, β-catenin was restricted to the perinuclear region in FHL2 KD cells. These data provide evidence that FHL2 is essential for promoting β-catenin nuclear enrichment in MM-MSCs. In conclusion, FHL2 is critical for Bzb-induced osteoblast differentiation of MM-MSCs and promotes the osteogenesis, through p53 signaling and β-catenin activation. Targeting FHL2 in MM may provide a new therapeutic strategy for treating MBD.

## Introduction

Multiple myeloma (MM)-related bone disease (MBD), characterized by lytic bone lesions, fracture, hypercalcemia, and severe pain, is the most common and life-threatening complication in MM patients (1, 2). The pathophysiology of MBD has been widely studied. It is well known that MBD results from complex cell interactions between MM malignant plasma cells (PCs) with bone cells, including osteocytes, osteoblasts, and osteoclasts (3). Suppressed osteoblast activity and increased osteoclast activity are two major aspects of MBD (4–6).

Bortezomib (Bzb), the first proteasome inhibitor (PI), which was approved for the treatment of MM, has been widely used and improved the survival of MM significantly. In addition to its anti-MM activity, preclinical and clinical data have shown that Bzb can inhibit osteoclast activity (7–10) and stimulate osteoblast activity (8–14), thus potentially exerting positive effects on bone metabolism (15, 16) independent of its effects on MM. Such induction of osteoblast differentiation may be mediated *via* β-catenin/T-cell factor TCF pathway and the modulation of Runx-2 (1, 17, 18). Further defining molecular mechanism of Bzb-enhanced bone formation in MM will be beneficial for the treatment of myeloma patients (5–8).

Four and a half LIM domains protein 2 (FHL2), a transcriptional coactivator and a scaffold protein that can interact with many proteins, including integrins and transcription factors in all compartments, has been reported to play a critical role in osteogenesis (19, 20). FHL2 overexpression upregulates the osteoblast differentiation markers and matrix mineralization. On the contrary, FHL2 deficiency inhibits osteoblast differentiation and bone formation (19). Here, we confirmed that Bzb promotes osteoblast differentiation of MM-MSCs and observed that FHL2 is upregulated during this process for the first time. While osteoblast differentiation of MM-MSCs was inhibited when FHL2 was knocked down (KD) by short hairpin RNA (shRNA). Further investigation showed that p53 signaling is associated with Bzb-induced FHL2 upregulation, and FHL2 promotes the nuclear enrichment of β-catenin in this process. These results suggest that FHL2 plays a critical role in the Bzb-induced osteoblast differentiation of MM-MSCs. Targeting FHL2 in MM may provide a new therapeutic strategy for treating MBD.

## Materials and Methods

### Cells

Primary MM cells were isolated from bone marrow (BM) aspirates of NDMM patients using Ficoll–Hypaque density gradient sedimentation and CD138 microbeads separation, following informed consent and institutional review board approval (the Institute of Hematology and Blood Diseases Hospital, Chinese Academy of Medical Sciences). CD138+ and CD138− cells were harvested separately for the following experiments. 293T cells (ATCC) cells were maintained in Dulbecco’s modified Eagle’s medium (DMEM, GIBCO, USA) with 10% fetal bovine serum (GIBCO, USA).

### Primary Bone Marrow-Derived MM-MSCs Culture

MSCs were derived from bone marrow of NDMM patients as described previously (21). Briefly, the CD138-negative mononuclear fraction from NDMM patients was cultured in DMEM with 10% fetal bovine serum for 24 h. Non-adherent cells were removed, and the culture medium was replaced with Mesen PRO RS Medium (GIBCO, USA) and refreshed twice a week thereafter. The cultures were maintained until 70% confluence. MSCs were selected by surface adherence and identified with cell markers (CD105, CD73, and CD90 positive, CD45, CD34, CD11b, CD19, and HLA-DR negative). The function of MSCs was further confirmed with osteoblasts and adipocytes differentiation *in vitro*. All the following assays were performed with the cells at passage 2 or 3.

### Osteogenic Differentiation of MM-MSCs

MM-MSCs were incubated in six-well microplates in the absence or presence of serial concentration of Bzb with osteogenic induction medium (OBI) for 21 days, and the medium was replaced every 3–4 days. The matrix mineralization was detected by Alizarin red staining. Briefly, cells were fixed with 10% formaldehyde for 15 min and stained with 40 mM Alizarin red (pH4.2) at room temperature for 20 min. To observe the calcium accumulation, images of cells were captured by the Nikon Eclipse 50i microscope (Nikon Inc., USA). Alkaline phosphatase index (API) is determined as described by Mukherjee (22).

### Knockdown Analysis

The MISSION TRC shRNA system (Sigma-Aldrich, USA) was used to generate stable gene KD of FHL2 according to the manufacturer’s protocol. Briefly, the lentiviral particles were harvested after transfection of the packaging 293T cells with the mixture of three lentiviral vectors using TurboFect (Fermentas, Canada). The stably transduced target MM-MSCs were obtained by screening against puromycin (Sigma-Aldrich, USA). The efficacy of KD was confirmed by quantitative polymerase chain reaction (qPCR) and Western blot. The validated clone sequence was CCGGCGACTGCTTTAACTGTAAGAACTCGAGTTCTTACAGTTAAAGCAGTCGTTTTT.

### Fluorescence *In Situ* Hybridization

Interphase fluorescence *in situ* hybridization (FISH) analysis was performed as described previously, on CD138-positive PCs and paired MM-MSCs from the same patient. The specific probes for FISH are as follows: del(13q) abnormality was assessed with LSI 13q34 (specific for the 13q34 locus, Abbott Laboratories); del(17p13) was assessed with LSI p53 (specific for the 17p13.1 locus, Abbott Laboratories); amp(1q21) was assessed with 1q21 (CKS1B) probe (GP Medical Technologies, Beijing, China); and IgH translocation was assessed with Vysis LSI IGH Dual Color, Break Apart Rearrangement Probe (Abbott Laboratories). If IgH translocation was positive, t(4;14) was assessed with LSI IGH/FGFR3 dual-color probe (Abbott Laboratories); t(11;14) was assessed with LSI IGH/CCND1 XT dual-color probe (Abbott Laboratories); and t(14;16) was assessed with IGH/MAF DF dual-color probe (Abbott Laboratories). A total of 200 interphase nuclei were analyzed. The cutoff values was set as recommended by the European Myeloma Network (EMN): 20% for deletions and numerical aberrations and 10% for translocations in the IgH locus and other translocations.

### Quantitative RT-PCR Analysis

Expression of human FHL2, TP53, Runx2, ALP, and Col1A1 transcript were determined using real-time quantitative reverse transcriptase–polymerase chain reaction (qPCR) based on TaqMan fluorescence methodology, following manufacturer protocols (Applied Biosystems, Foster City, CA). Relative expression was calculated using the comparative ΔΔ(Ct) method.

### Western Blot

Cells were harvested and lysed in lysis buffer (RIPA, Boston Bio Products) supplemented with Halt protease and phosphatases inhibitor cocktail (Thermo fisher Scientific). Protein concentrations were determined using Pierce™ BCA Protein Assay Kit (Thermo fisher Scientific). Equal amounts of protein (20–30 µg) were loaded on 10% or 4-12% Bis–Tris gel and transferred to PVDF or NC membrane followed by immunoblotting with 5% milk. Then, membranes were incubated with first antibody at 4°C overnight. After washed with Tris-buffered saline with Tween 20 (TBST) three times for 5 min, membranes were incubated with horseradish peroxidase (HRP)-conjugated second antibody for 1 h at room temperature, washed again with TBST three times for 5 min.

### Immunofluorescent Staining

Immunostaining cells were cultured on 8 mm × 8 mm glass slide in a 24-well plate under the presence or absence of 2 nM Bzb. After the treatment, cells were fixed in 4% paraformaldehyde, permeabilized with 0.5% Triton-X 100, incubated with anti-β-catenin primary antibody (Santa Cruz, USA) overnight, and stained with Cy3-labeled secondary antibody (Invitrogen, USA) and 4′,6-diamidino-2-phenylindole (DAPI) (Vysis, USA). The fluorescence was observed under a Leica confocal laser microscope.

### Statistical Analysis

The data were expressed as mean ± standard deviation (SD) over three independent experiments. Statistical significance of differences was analyzed by t-test for comparison between two groups or one-way ANOVA for comparison among more than two groups using the GraphPad Prism 8 software. p < 0.05 by two-tailed test was considered as statistically significant.

## Results

### FHL2 Is Upregulated During Bzb-Induced Osteoblast Differentiation of MM-MSCs

**Figure 1:**
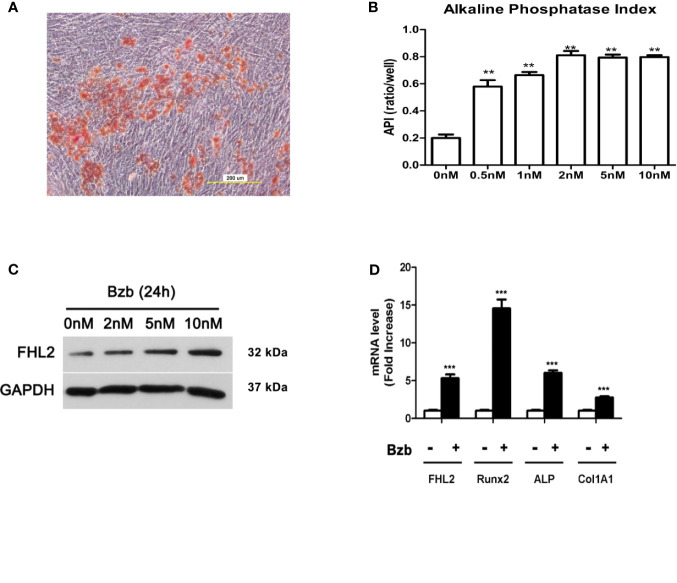
FHL2 expression is upregulated during Bzb-induced osteoblast differentiation of MM-MSCs. **(A)** Alizarin red staining was performed to show the osteoblast activity of differentiated MM-MSCs. **(B)** MM-MSCs were treated with indicated concentrations of Bzb for 7 days, and alkaline phosphatase index (API) was measured. **(C)** The protein level of FHL2 was evaluated by Western blot analysis after 24 h at indicated concentrations of Bzb. **(D)** FHL2 and osteoblast markers Runx2, ALP, and Col 1A1 mRNA expression was determined by quantitative RT-PCR at day 7. Results are expressed as mean ± SD (n = 3). **p < 0.01 *vs*. controls, ***p < 0.001 *vs*. controls.

To investigate the role of FHL2 in Bzb-induced osteogenic differentiation of MM-MSCs, totally 12 samples were used for the MM-MSCs isolation. CD138-negative mononuclear fraction was isolated from bone marrow of NDMM patients, and MM-MSCs were cultured. The purity of MSCs was about 95%, confirmed by flow cytometry (**Supplementary Figure S1**). Functional studies of osteoblast and adipocytes differentiation *in vitro* were confirmed with Alizarin red staining (**Figure 1A**) and Oil Red O Staining (**Supplementary Figure S2**), respectively. Osteoblast was induced in six-well microplates in the presence or absence of 2 nM Bzb for 21 days, and the medium was replaced every 3–4 days. Alkaline phosphatase index (API) was measured after 7 days of treatment. As shown in **Figure 1B**, API increased at a dose-dependent manner to Bzb until 2 nM. Western blot analysis showed that FHL2 expression was also upregulated with Bzb treatment in a dose-dependent manner (**Figure 1C**). Quantitative real-time PCR (RT-PCR) analysis showed that messenger RNA (mRNA) levels of FHL2 and the main osteoblast markers Runx2, ALP, and Col1A1 were upregulated with 7 days treatment of Bzb at 2 nM (**Figure 1D**). These data confirm that Bzb promotes MM-MSCs osteoblast differentiation, bone formation, and regeneration. Meanwhile, these data also indicate that FHL2 expression is increased during Bzb-induced osteoblast differentiation of MM-MSCs.

### Knocking Down of FHL2 Inhibits Bzb-Induced Osteoblast Differentiation of MM-MSCs

**Figure 2:**
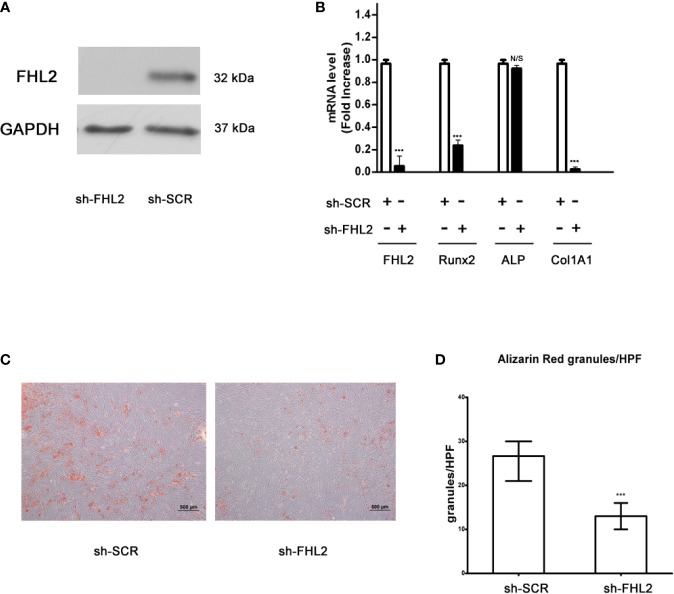
Knocking down of FHL2 impairs Bzb-induced osteoblast differentiation of MM-MSCs. MM-MSCs were infected with shFHL2 and shSCR. **(A)** FHL2 protein levels were determined by Western blot. **(B)** Infected cells were cultured for 7 days with 2 nM Bzb with osteogenic induction medium (OBI). mRNA levels of FHL2 and osteoblast markers Runx2, ALP, and Col1A1 by quantitative RT-PCR. **(C, D)** Infected cells were cultured for 21 days with 2 nM Bzb with osteogenic induction medium (OBI) and 21 days. Alizarin red staining **(C)** was performed, and alkaline phosphatase index (API) **(D)** was determined. Results are expressed as mean ± SD (n = 3). ***p < 0.001 *vs* controls; N/S, not significant. Scale bars = 500 µm.

To confirm the role of FHL2 in the induction of MSCs differentiation into osteoblasts, we used pLKO.1 lentiviral shRNA vector (pLKO.1-FHL2, shFHL2) to knock down the expression of FHL2 in MM-MSCs. Silencing of FHL2 was confirmed at both mRNA and protein levels (**Figures 2A, B**). Compared with the control cells (infected with shSCR), the mRNA levels of osteoblast marker genes, including Runx2 and Col1A1, were also downregulated significantly by FHL2 KD (**Figure 2B**), supporting the role of FHL2 in osteogenic differentiation of MSCs. In addition, the impaired osteogenic capacity of MM-MSCs by FHL2 KD was further confirmed by Alizarin red staining of the mineralization (**Figures 2C, D**). These results demonstrate that FHL2 is required for maintenance of the osteogenic differentiation potential of MM-MSCs.

### p53 Signaling Is Associated With Bzb-Induced FHL2 Upregulation

The potential mechanisms through which FHL2 promotes the Bzb-induced osteogenic differentiation of MM-MSCs was then investigated. It has been reported that cells with wild-type (WT) TP53 exhibit higher level of FHL2 mRNA (23). Therefore, we investigated whether p53 related to FHL2 in Bzb-induced osteogenesis of MM-MSCs. Notably, chromosomal abnormalities including deletion of WT TP53 and RB-1 are important cytogenetic clonal markers in malignant PCs of MM (24). Interphase FISH with probes for 13q14 (LSI RB-1), 14q32 (LSI IGHC/IGHV), 1q21(LSI CKS1B), and 17p13(LSI TP53) were performed in paired CD138-positve PCs and CD138-negative MM-MSCs (n = 6). As shown in **Supplementary Figure S3** and **Supplementary Table S1**, MM-MSCs do not carry the same chromosomal abnormalities as their PC counterparts, and all the MM-MSCs harbor two copies of TP53.

**Figure 3:**
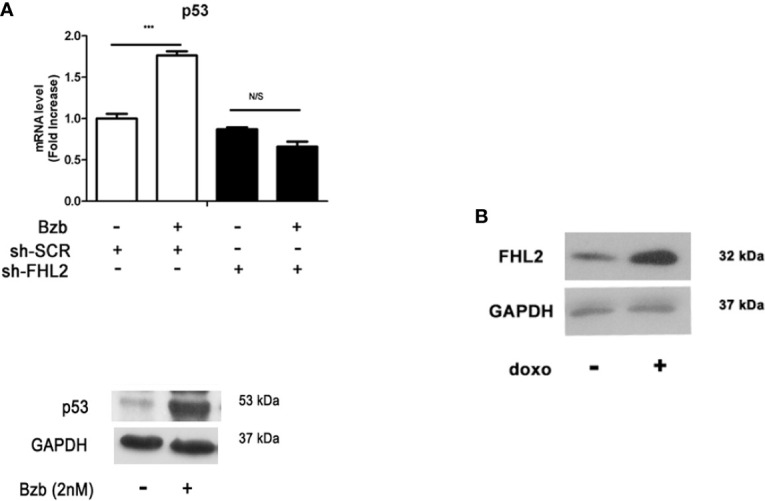
p53 signaling is associated with Bzb-induced FHL2 upregulation. **(A)** MM-MSCs infected with shFHL2 and shSCR were differentiated in the presence or absence of Bzb (2 nM) for 7 days; the mRNA levels of TP53 were assessed by quantitative RT-PCR (up) and Western blot (blow). **(B)** The protein level of FHL2 was measured after 1 µg/ml doxorubicin (doxo) treatment for 24 h. Results are expressed as mean ± SD (n = 3). ***p < 0.001 *vs*. controls; N/S, not significant.

We further examined the expression levels of TP53 in Bzb-treated MM-MSCs in different FHL2 background. mRNA level of TP53 was upregulated significantly with Bzb treatment in MM-MSCs with WT FHL2 (**Figure 3A**), which is consistent with the increased expression of FHL2, as shown previously (**Figures 1C, D**). However, this phenotype was abolished with FHL2 KD. The protein level of p53 were also upregulated with Bzb treatment in MSCs with WT FHL2 (**Figure 3B**). To confirm that FHL2 expression upregulation is related to p53, but not proteasome inhibition, we treated cells with p53-inducing agent, doxorubicin. As expected, FHL2 was also upregulated significantly (**Figure 3B**). These results suggest that p53 signaling involves in the Bzb-induced FHL2 upregulation during Bzb-induced osteoblast differentiation of MM-MSCs.

### FHL2 Promotes the Nuclear Enrichment of β-Catenin

**Figure 4:**
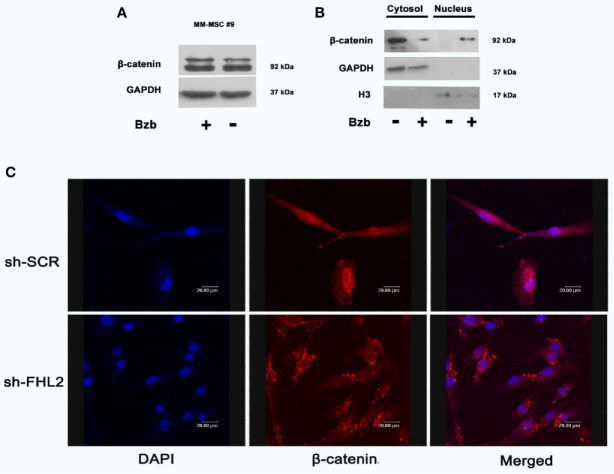
FHL2 is necessary for Bzb-induced β-catenin nuclear enrichment in MM-MSCs. Cells were treated with 2 nM Bzb for 24 h, and the expression of β-catenin was measured by Western blot **(A, B)**. Immunofluorescence microscopy with antibody specific for β-catenin (red) and nuclei (DAPI, blue) were used to show the localization **(C)**. Scale bars = 20 µm. OBI, osteogenic induction medium.

Considering that β-catenin signal pathway plays an important role in the proliferation and differentiation of osteoblasts (25–27), the protein expression levels of β-catenin were measured. Results from Western blot showed total β-catenin protein was not changed significantly with and without Bzb treatment (**Figure 4A**). We then surveyed the subcellular localization of β-catenin. As shown in **Figure 4B**, β-catenin protein was mostly enriched in nucleus after Bzb treatment. However, Bzb-induced nuclear enrichment of β-catenin was impaired with FHL2 KD β-Catenin was restricted to the perinuclear region in FHL2 KD cells (**Figure 4C**). These results imply that FHL2 promotes the nuclear enrichment of β-catenin during Bzb-induced osteoblast differentiation of MM-MSCs.

## Discussion

Bzb, a first-in-class proteasome inhibitor, can induce osteoblast differentiation of MSCs *in vitro* and in animal models *in vivo* (18, 22) and has been related to the biochemical markers of bone remodeling in MM patients (11–14, 28). The molecular mechanisms by which Bzb enhances the differentiation of MSCs toward osteoblasts are not fully understood. Studies have demonstrated that this effect is mediated by stabilizing the activities of critical transcription factors required for osteoblast differentiation, such as Runx-2 and β-catenin (18, 22). The present study has shown for the first time that FHL2, another important transcription factor, plays an critical role in the Bzb-induced osteoblast differentiation of MM-MSCs and may thus represent a new target for the efficient promotion of bone regeneration.

FHL2 is a multifunctional adaptor protein that can interact with numerous molecules in different organelles, involve in various functional activities, often opposing functions, including cell proliferation, apoptosis, adhesion, migration, structural stability, gene expression, and even carcinogenesis (23, 29–43). The great capacity of FHL2 to bind diverse proteins, and its versatile functions, have been the topics of many investigations. Hamidouche et al. have shown that forced expression of FHL2 itself is sufficient to promote the osteogenic differentiation of murine and human MSCs (20). Notably, they also found that FHL2 play an important role in the osteoblast differentiation during dexamethasone treatment (20). However, whether FHL2 mediate Bzb-induced osteogenic differentiation of MM-MSCs was unknown. In this report, we observed the increased expression of osteoblast markers (Runx2, ALP, and Col1A1) with Bzb treatment, which confirmed that Bzb promotes osteogenic differentiation of MM-MSCs. Meanwhile, FHL2 was also upregulated during this process. FHL2 KD with shRNA reduced the expression of osteoblast marker genes and blocked the osteogenic differentiation of MM-MSCs regardless the presence or absence of Bzb, implying that FHL2 is an important activator of the osteogenic differentiation of human MSCs under a proteasome inhibition condition.

**Figure 5:**
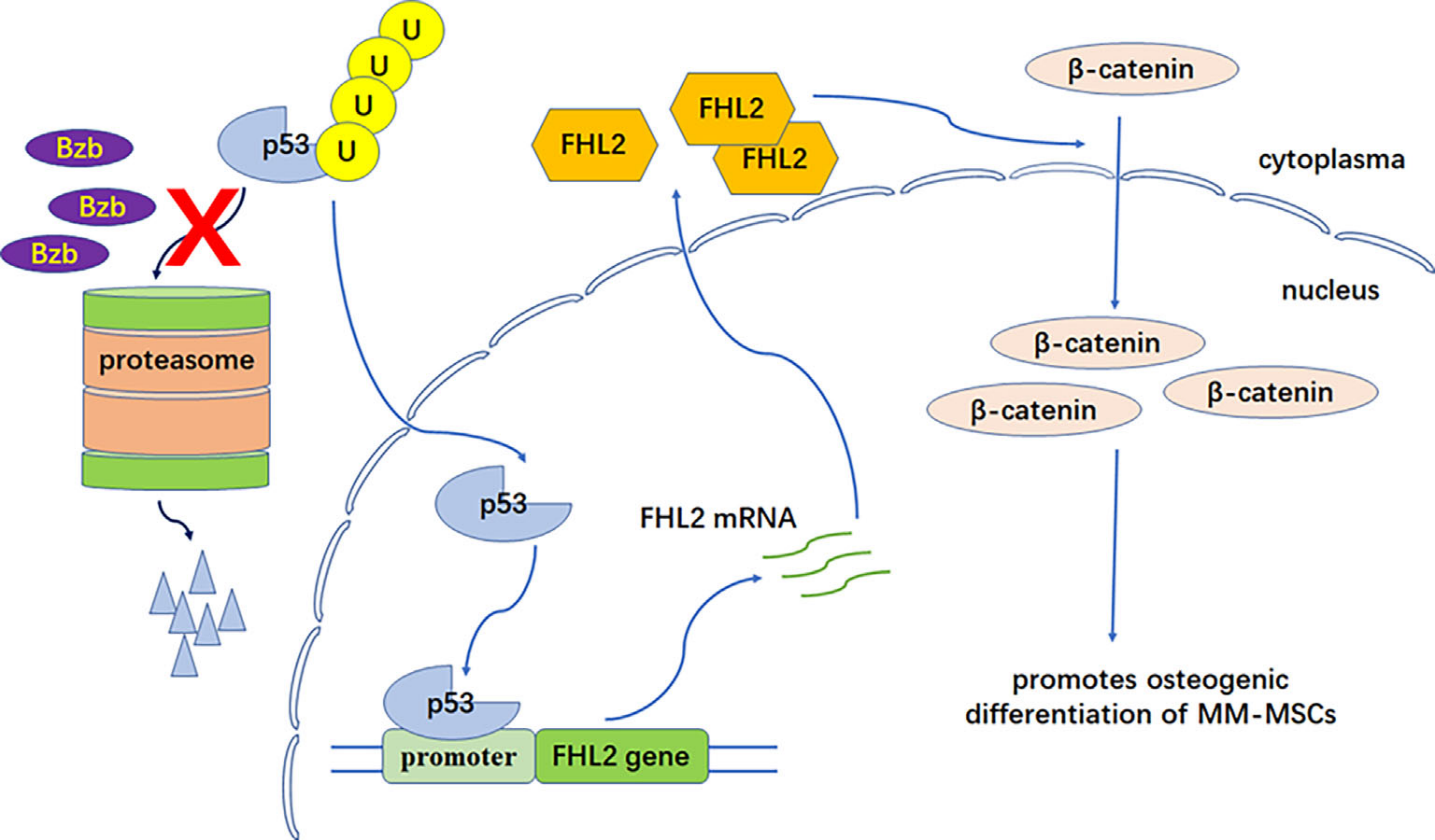
FHL2 functional model in Bzb-induced osteoblast differentiation of MM-MSCs. P53 degradation is weakened with proteasome inhibition by Bzb. Upregulated p53 binds to the binding motifs in the promoter region of the FHL2 gene and promotes FHL2 expression. Finally, increased FHL2 changes subcellular localization of β-catenin (nuclear enrichment) and promotes osteogenic differentiation of MM-MSCs.

It was putative that there is possible p53 transcription factor binding motifs in promoter region of FHL2. Although the actual contribution of p53 has not been fully investigated, some evidence has shown that it may be involved in the transcription of the FHL2 gene (23). We hypothesized that Bzb-induced FHL2 upregulation was mediated by p53. We showed here that the expression level of p53 was upregulated significantly in MM-MSCs with Bzb treatment, which can be partly explained by p53 degradation inhibition with Bzb. In addition, FHL2 was also upregulated significantly with p53-inducing agent, doxorubicin, which confirmed that FHL2 upregulation is related to p53 but not proteasome inhibition. These data imply that Bzb-induced FHL2 upregulation is mediated at least partly through p53 signaling. p53 degradation is weakened with proteasome inhibition by Bzb. Upregulated p53 binds to the binding motifs in the promoter region of FHL2 gene and promotes FHL2 expression (**Figure 5**). Further studies are needed to confirm this hypothesis and to make it clear whether other molecules in the p53 pathway are involved in bortezomib-induced FHL2 expression.

We then investigated the potential mechanisms through which FHL2 promotes Bzb-induced osteoblast differentiation in MM-MSCs. It is widely accepted that osteoblast activity is mediated mainly by Wnt/β-catenin pathway. The suppression of Wnt/β-catenin signaling pathway is reportedly related to the osteolytic bone disease in MM, and the emerging data suggest that Bzb induces osteogenic differentiation *via* activation of β-catenin (18, 44–46). It is reasonable to explore the role of β-catenin in the Bzb-induced osteogenic differentiation. Unlike the previous report (18), we did not find significant change at protein level of total β-catenin protein with or without Bzb treatment. However, it was mostly enriched to nuclei in MSCs after Bzb treatment. On the contrary, β-catenin was distributed in the cytoplasm in FHL2 KD cells. These data provide evidence that FHL2 is essential for promoting β-catenin nuclear enrichment in MSCs. It is our limitation for not studying the interaction between FHL2 and β-catenin. A previous report showed that FHL2 and β-catenin coimmunoprecipitated in both the cytosolic and nuclear fractions, suggesting physical interactions between the two proteins in C3H10T1/2 MSCs (20). We will focus on this point for our further study.

In summary, this study implies that FHL2 plays an important promoting role in Bzb-induced osteoblast differentiation of MM-MSCs. p53 degradation is weakened with proteasome inhibition by Bzb. Upregulated p53 binds to the binding motifs in the promoter region of FHL2 gene and promotes FHL2 expression. Finally, increased FHL2 changes subcellular localization of β-catenin (nuclear enrichment) and promotes osteogenic differentiation of MM-MSCs (**Figure 5**). Moreover, FHL2 has been reported to attenuate the TRAF6-mediated RANK signaling and antiosteoclastogenic effect (47–49). Therefore, FHL2 may play an important role in the Bzb-induced bone remodeling in MM. Proteasome inhibition therapy and treatment targeting FHL2 in the bone microenvironment may thus provide a practical and specific treatment for MBD.

## Data Availability Statement

The original contributions presented in the study are included in the article/**Supplementary Material**. Further inquiries can be directed to the corresponding authors.

## Author Contributions

ZX, YX, and LQ designed the study and wrote the manuscript. ZX, XW, MH, and ZY performed the experiments. YX, GA, and LQ performed patients recruitment and samples collection. ZX, YX, GA, and LQ analyzed the data. All authors contributed to the article and approved the submitted version.

## Funding

This work was supported by The Natural Science Foundation of China (NSFC, 81101794, 81570181, and 81920108006); the Chinese Academy of Medical Sciences (CAMS) Innovation Fund for Medical Sciences CAMS-2017-I2M-1-005 and 631 2016-I2M-3-023; and the Fundamental Research Funds for the Central Universities (3332020055).

## Conflict of Interest

The authors declare that the research was conducted in the absence of any commercial or financial relationships that could be construed as a potential conflict of interest.

## Publisher’s Note

All claims expressed in this article are solely those of the authors and do not necessarily represent those of their affiliated organizations, or those of the publisher, the editors and the reviewers. Any product that may be evaluated in this article, or claim that may be made by its manufacturer, is not guaranteed or endorsed by the publisher.
