# Supplementary figures and images for: Four and a Half LIM Domains Protein 2 Mediates Bortezomib-Induced Osteogenic Differentiation of Mesenchymal Stem Cells in Multiple Myeloma Through p53 Signaling and β-Catenin Nuclear Enrichment
Source: Front Oncol. 2021 Sep 13;11:729799. doi: 10.3389/fonc.2021.729799 (PMC8473907; doi:10.3389/fonc.2021.729799)

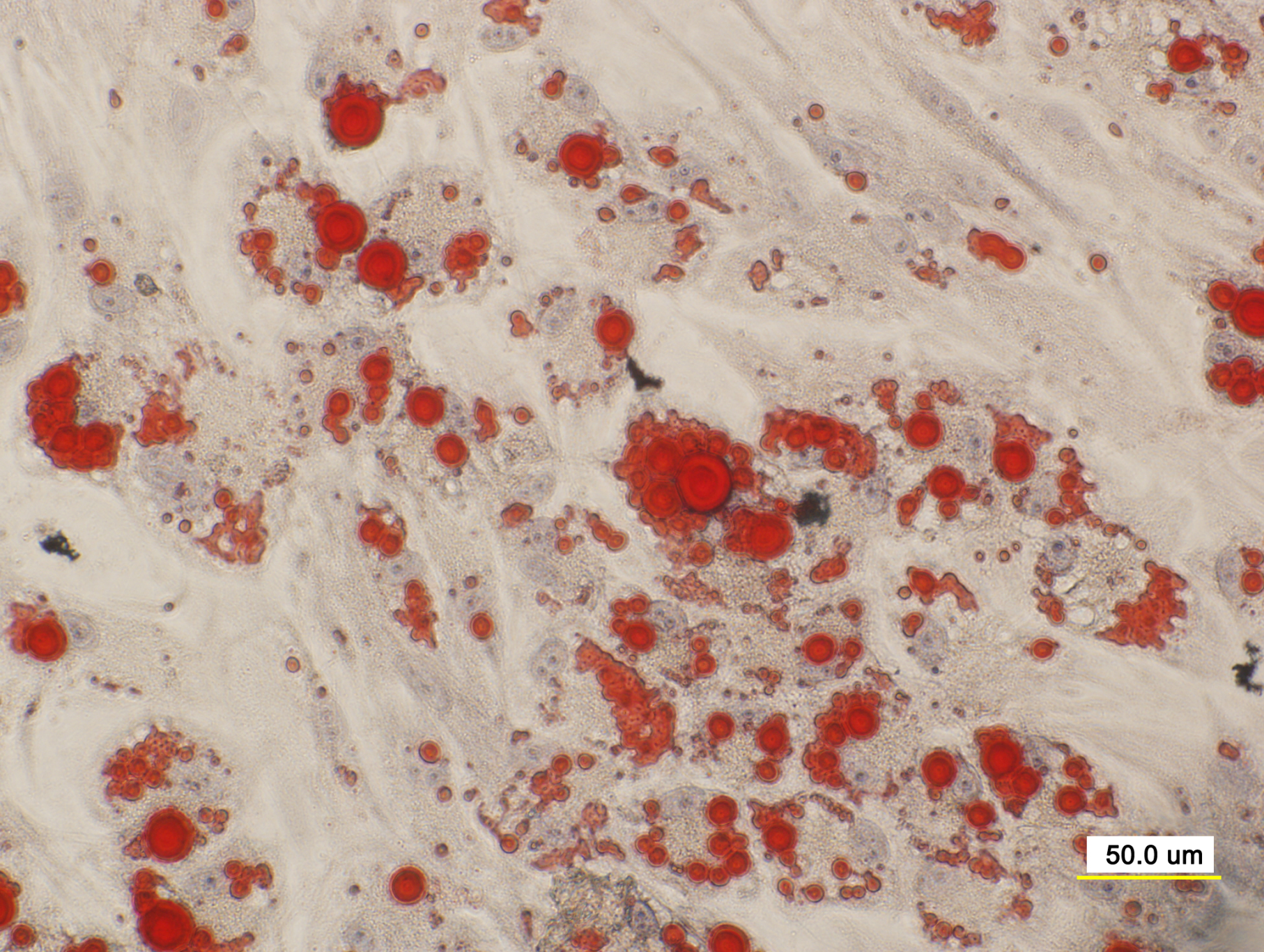

50.0 um

Supplement: Supplementary file 1 [file DataSheet_1.pdf]

A

PC

MSC

p53 deletion  
patient 1

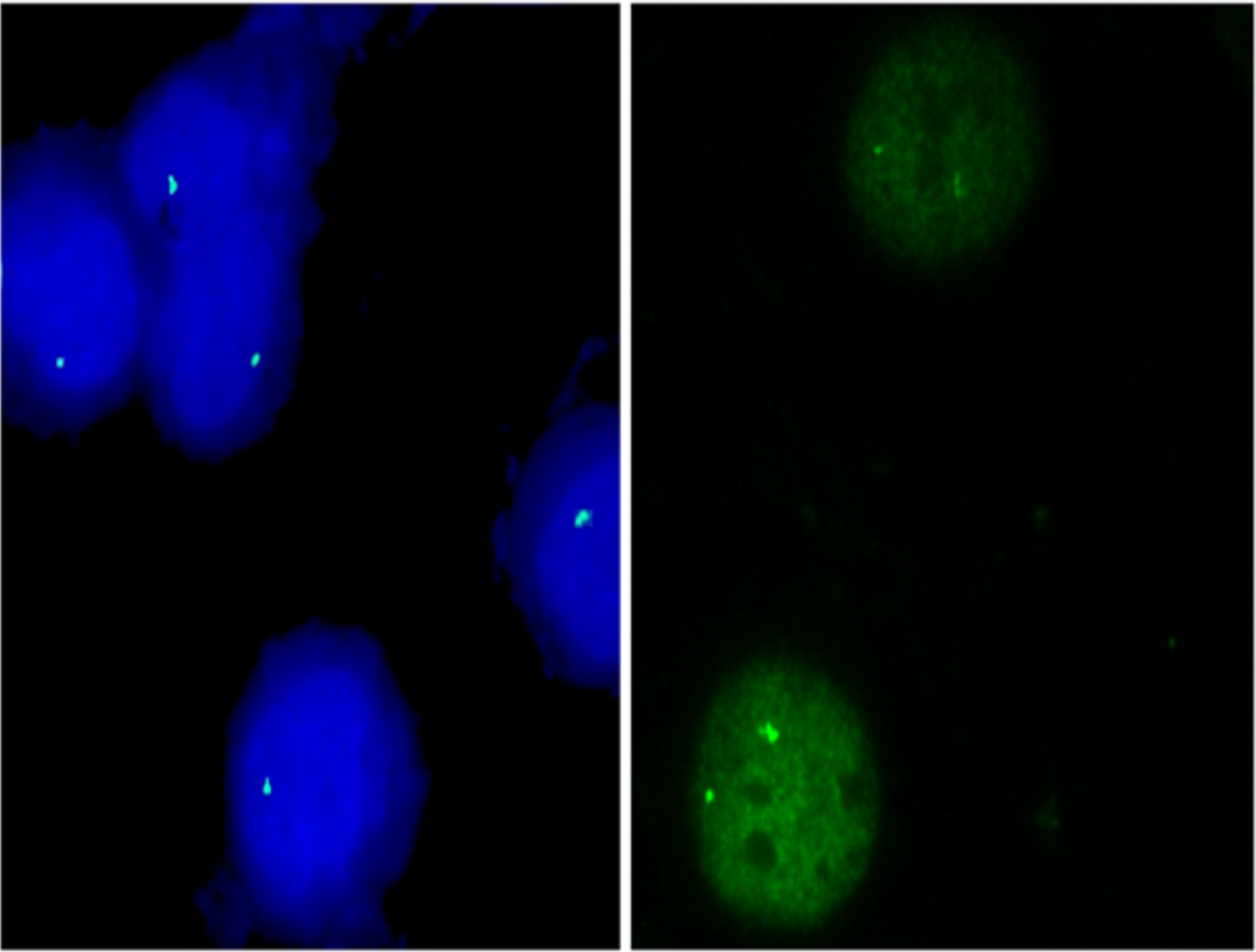

q21 amplicaiton  
patient 5

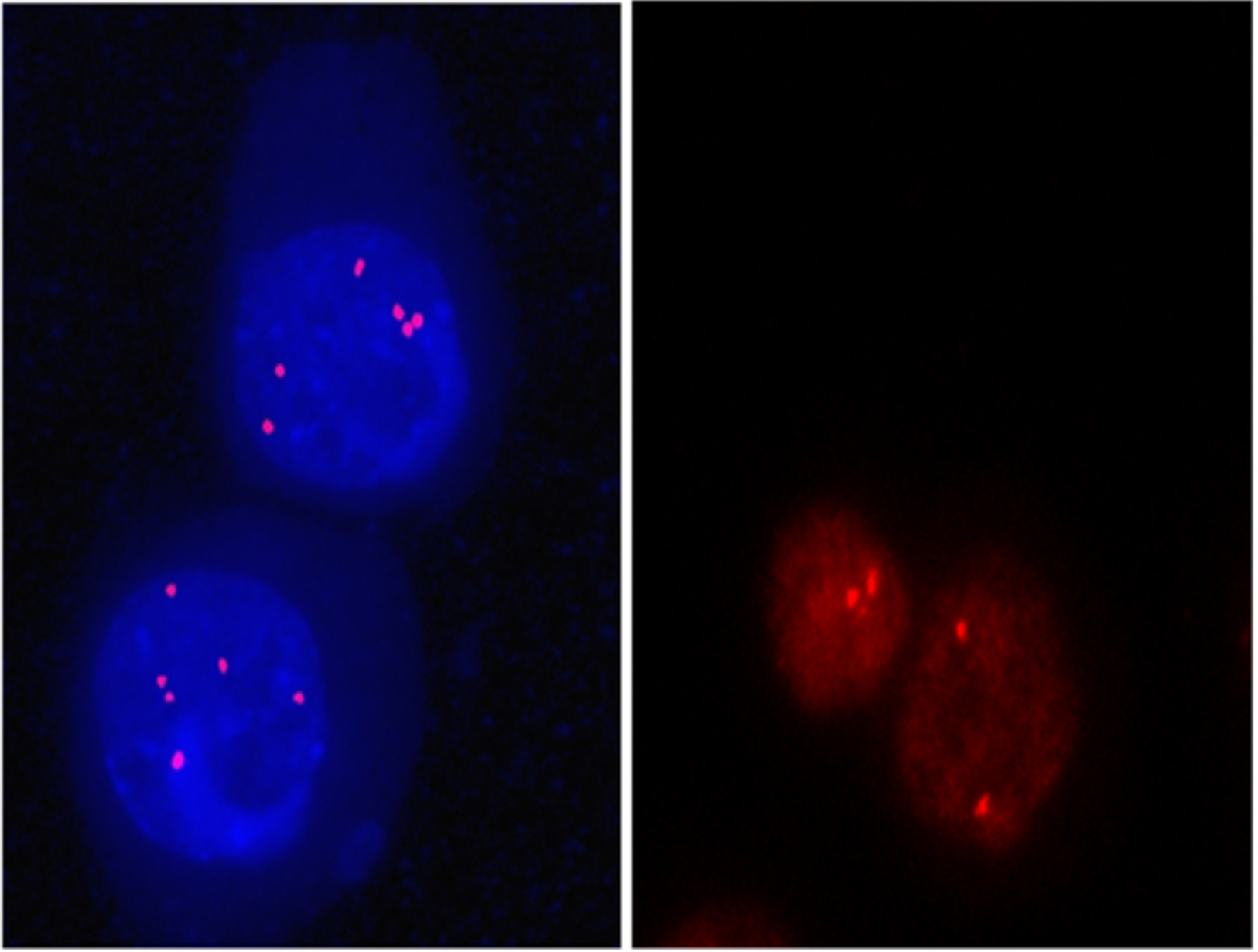

RB deletion  
patient 3

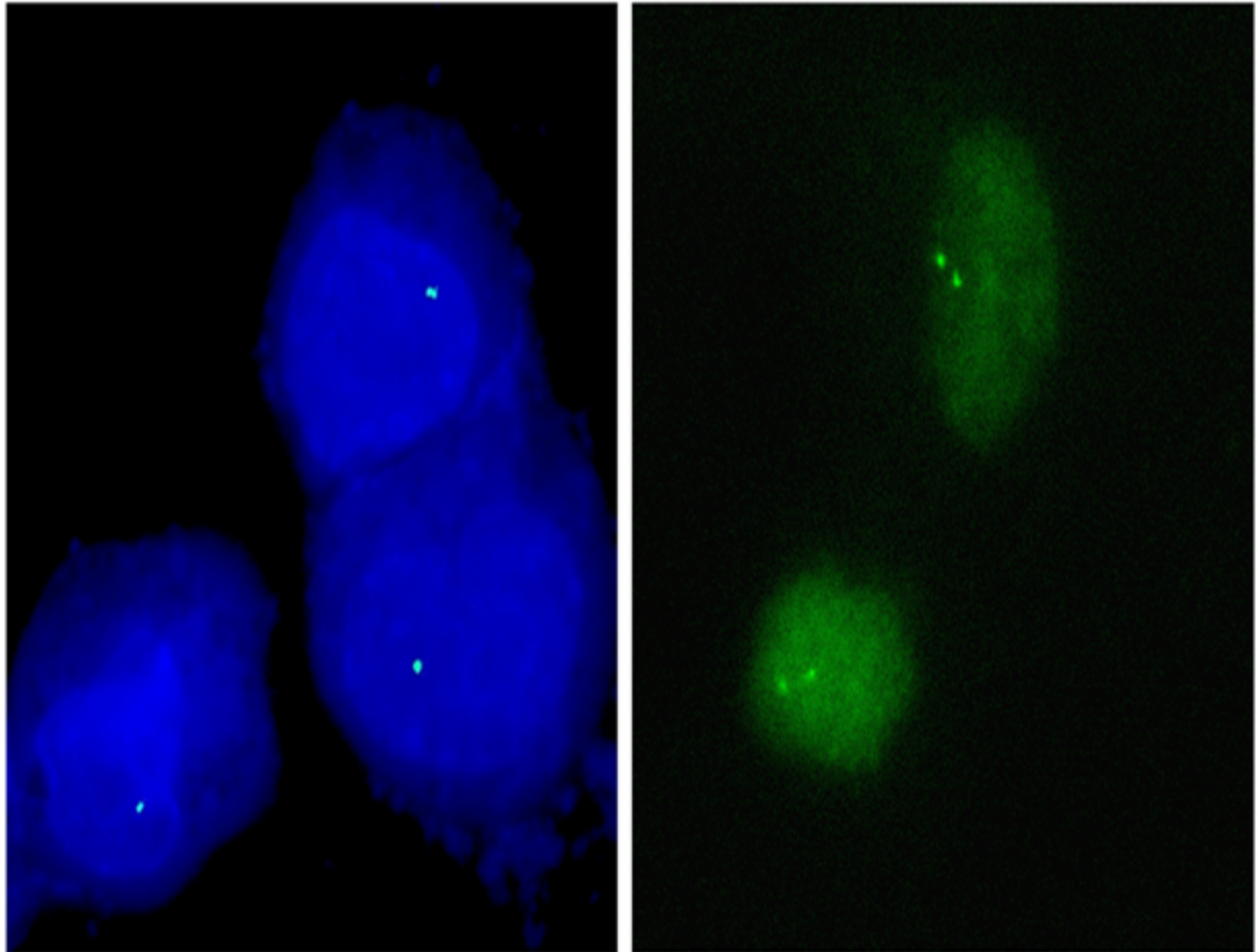

IGHC/IGHV  
patient 4

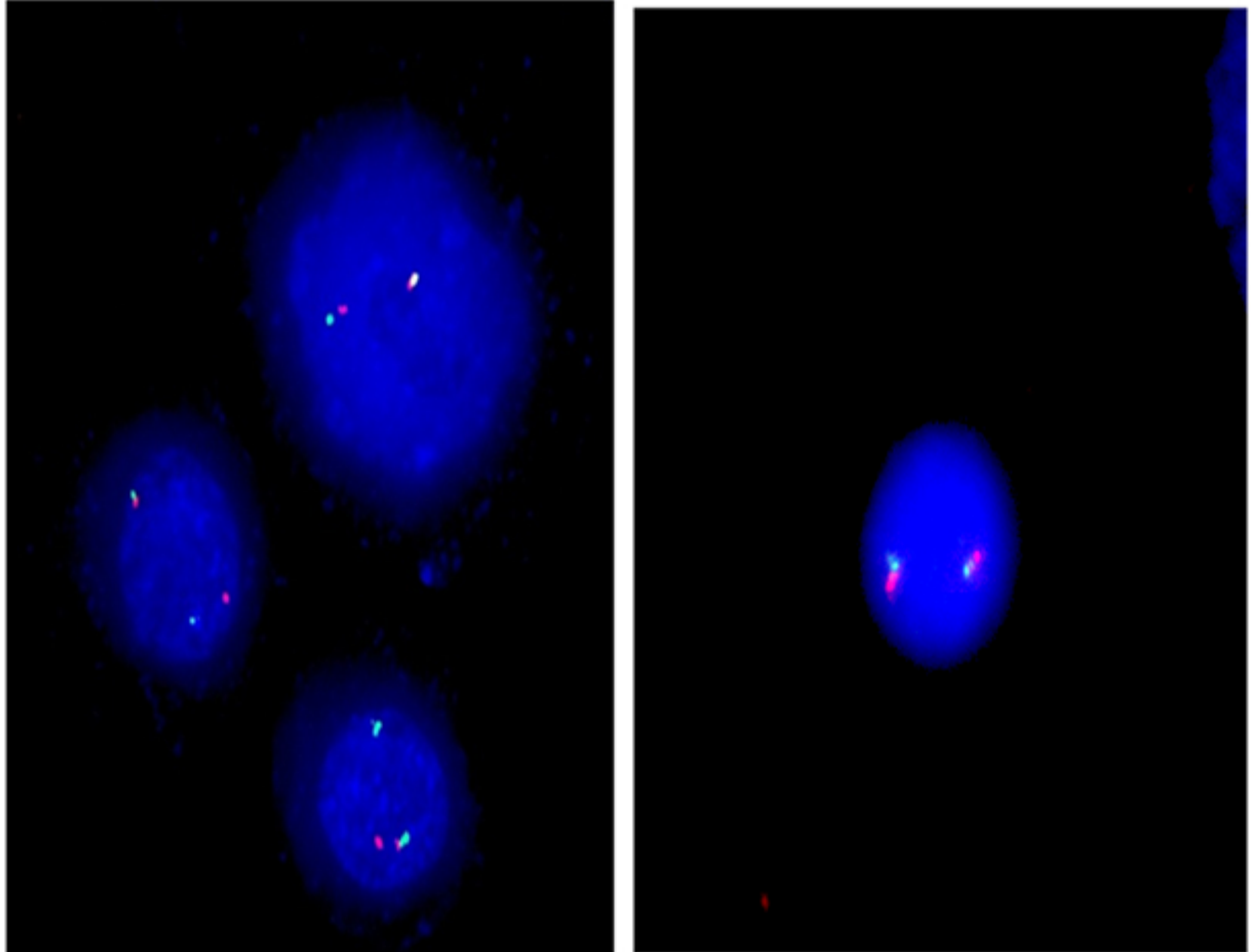

Supplement: Supplementary file 2 [file DataSheet_2.pdf]
