# Supplementary material for: Four and a Half LIM Domains Protein 2 Mediates Bortezomib-Induced Osteogenic Differentiation of Mesenchymal Stem Cells in Multiple Myeloma Through p53 Signaling and β-Catenin Nuclear Enrichment
Source: Front Oncol. 2021 Sep 13;11:729799. doi: 10.3389/fonc.2021.729799 (PMC8473907; doi:10.3389/fonc.2021.729799)
Supplement: Supplementary file 3 [file DataSheet_3.pdf]

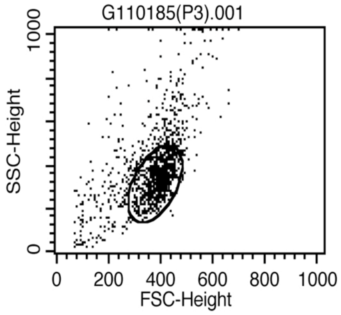


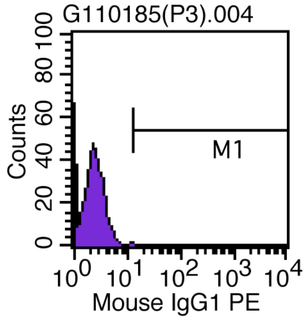


| Marker | % Gated |
|---|---|
| All | 100.00 |
| M1 | 0.00 |

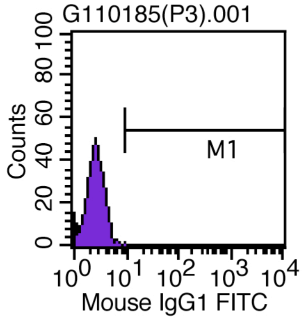


| Marker | % Gated |
|---|---|
| All | 100.00 |
| M1 | 0.00 |

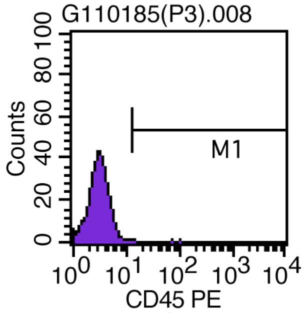


| Marker | % Gated |
|---|---|
| All | 100.00 |
| M1 | 0.13 |

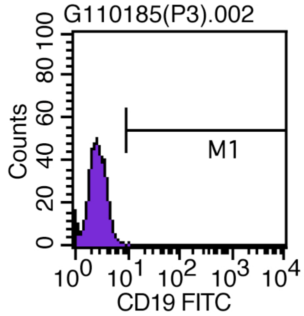


| Marker | % Gated |
|---|---|
| All | 100.00 |
| M1 | 0.03 |

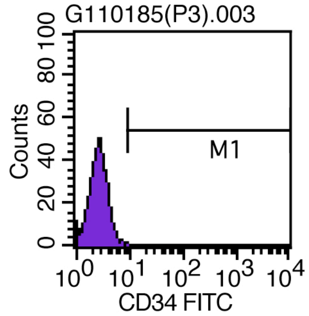


| Marker | % Gated |
|---|---|
| All | 100.00 |
| M1 | 0.00 |

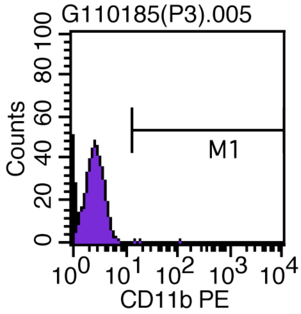


| Marker | % Gated |
|---|---|
| All | 100.00 |
| M1 | 0.09 |

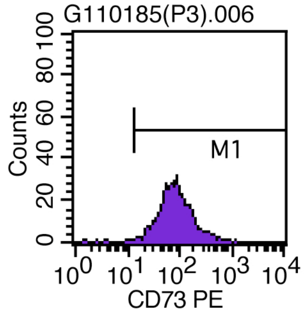


| Marker | % Gated |
|---|---|
| All | 100.00 |
| M1 | 99.69 |

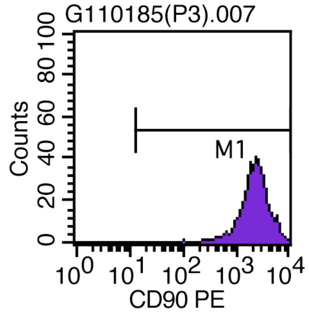


| Marker | % Gated |
|---|---|
| All | 100.00 |
| M1 | 100.00 |

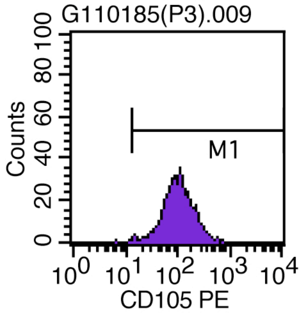


| Marker | % Gated |
|---|---|
| All | 100.00 |
| M1 | 99.88 |

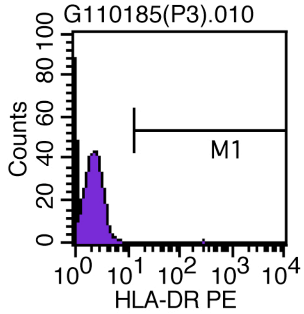


| Marker | % Gated |
|---|---|
| All | 100.00 |
| M1 | 0.03 |

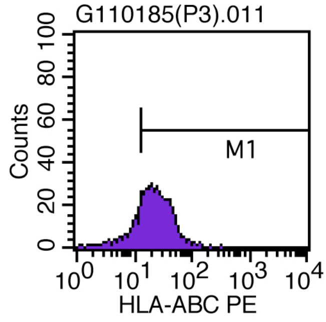


| Marker | % Gated |
|---|---|
| All | 100.00 |
| M1 | 82.23 |
