# Supplementary material for: Four and a Half LIM Domains Protein 2 Mediates Bortezomib-Induced Osteogenic Differentiation of Mesenchymal Stem Cells in Multiple Myeloma Through p53 Signaling and β-Catenin Nuclear Enrichment
Source: Front Oncol. 2021 Sep 13;11:729799. doi: 10.3389/fonc.2021.729799 (PMC8473907; doi:10.3389/fonc.2021.729799)
Supplement: Supplementary file 4 [file DataSheet_4.pdf]

Supplemental table 1. Genetic aberrations found in paired MSCs and PCs of MM patients.

**Patients ID number**	**Cell type**	**p53 deletion**	**1q21 amplification**	**RB-1 deletion**	**D13S319 deletion**	**IGH translocation**
**1**	MSC	-	-	-	-	-
	PC	-	+	-	-	-
**2**	MSC	-	-	-	-	-
	PC	-	-	+	+	-
**3**	MSC	-	-	-	-	-
	PC	-	-	+	+	-
**4**	MSC	-	-	-	-	-
	PC	-	-	-	-	+
**5**	MSC	-	-	-	-	-
	PC	-	+	-	-	-
**6**	MSC	-	-	-	-	-
	PC	+	-	-	-	-

PC, plasma cells; MSC, mesenchymal stem cell; IGH, immunoglobulin heavy chain. Interphase fluorescence in situ hybridization (FISH) analysis was performed.

Supplementary Figure 1. The detailed characteristics of MM-MSCs. As measured by flow cytometry, all the MM-MSCs expressed CD73, CD90 and CD105 (95%). In addition, these cells lacked expression of CD14, CD19, CD34, CD45 and HLA-DR (≤2%).

Supplementary Figure 2. Adipocytes differentiation of MM-MSCs in vitro. Oil Red O staining was performed to demonstrate the adipocytes differentiation of MM-MSCs.

Supplementary Figure 3. Genetic aberrations detected by FISH in paired MSCs and PCs of MM patients. Interphase fluorescence in situ hybridization (FISH) analysis was performed in MM-MSCs and its paired CD138+ plasma cells from MM patients.
